# Impact of first wave of SARS-CoV-2 infection in patients with Systemic Lupus Erythematosus: Weighting the risk of infection and flare

**DOI:** 10.1371/journal.pone.0245274

**Published:** 2021-01-13

**Authors:** Dina Zucchi, Chiara Tani, Elena Elefante, Chiara Stagnaro, Linda Carli, Viola Signorini, Francesco Ferro, Francesca Trentin, Giovanni Fulvio, Chiara Cardelli, Marco Di Battista, Gianmaria Governato, Antonio Figliomeni, Marta Mosca

**Affiliations:** Rheumatology Unit, Department of Clinical and Experimental Medicine, University of Pisa, Pisa, Italy; National Institute for Infectious Diseases Lazzaro Spallanzani-IRCCS, ITALY

## Abstract

**Introduction:**

The aim of this study was to investigate the incidence and clinical presentation of SARS-CoV-2 infections in a Systemic Lupus Erythematosus (SLE) cohort; to assess correlations with disease characteristics and rheumatic therapy; and to evaluate the occurrence of treatment discontinuation and its impact on disease activity.

**Materials and methods:**

SLE patients monitored by a single Italian centre were interviewed between February and July 2020. Patients were considered to be positive for SARS-CoV-2 infections in case of 1) positive nasopharyngeal swab; 2) positive serology associated with COVID19 suggesting symptoms. The following data were also recorded: clinical symptoms, adoption of social distancing measures, disease activity and treatment discontinuation.

**Results:**

332 patients were enrolled in the study. Six patients (1.8%) tested positive for SARS-CoV-2 infection, with the incidence being significantly higher in the subgroup of patients treated with biological Disease-Modifying Anti-Rheumatic Drugs (p = 0.005), while no difference was observed for other therapies, age at enrollment, disease duration, type of cumulative organ involvement or adoption of social isolation. The course of the disease was mild. Thirty-six patients (11.1%) discontinued at least part of their therapy during this time period, and 27 (8.1%) cases of disease flare were recorded. Correlation between flare and discontinuation of therapy was statistically significant (p<0.001). No significant increase of rate of flare in a subgroup of the same patients during 2020 was observed.

**Conclusion:**

Treatment discontinuation seems to be an important cause of disease flare. Our findings suggest that abrupt drug withdrawal should be avoided or evaluated with caution on the basis of individual infection risk and comorbidities.

## Introduction

Severe acute respiratory coronavirus 2 (SARS-CoV-2) and related coronavirus disease-2019 (COVID19) represent a serious issue to public health, while data concerning SARS-CoV-2 infections in patients affected by Systemic Lupus Erythematosus (SLE) remain scarce [1–3].

Patients with SLE represent an interesting study group due to being frequently exposed both to factors that may increase the risk of viral infections and related complications, and to drugs that may improve the outcome. Indeed, alterations in the immune response and immunosuppressive drugs make patients susceptible to viral infections [4], while glucocorticoids (GCs), chloroquine and hydroxychloroquine (HCQ) were proposed among the treatments to improve outcomes in COVID19 patients [5–7].

The aims of this study were: firstly, to investigate the incidence and clinical presentation of SARS-CoV-2 infections in a SLE cohort monitored by a single Italian centre; secondly, to assess correlations with disease characteristics and anti-rheumatic therapies; thirdly, to evaluate the occurrence of treatment discontinuation and its impact on disease activity; finally, to compare the flare rate of the cohort in the pandemic season with that of the equivalent period in 2019.

Based on previous reports, our hypothesis is that COVID-19 incidence and course is similar in SLE patients compared to other chronic diseases while personal and societal choices regarding the health emergency could have a significant impact on disease outcomes at least in the short term.

## Materials and methods

This cross-sectional study involved adult patients with SLE satisfying the 2019 European League Against Rheumatism/American College of Rheumatology classification criteria [8], regularly monitored by the Lupus Clinic of the University of Pisa.

Patients were interviewed during telemedicine visits between February and July 2020 concerning any possible symptoms in the same time period suggesting SARS-CoV-2 infections, results of nasopharyngeal swab and serological test if available, close contacts with confirmed cases, adoption of social distancing measures, concomitant medications and adherence to them, and cases of disease flare. In the case of confirmed infections, we collected data regarding clinical course, need for hospitalisation and requirement for therapy or ventilatory support.

We considered cases with positive nasopharyngeal swab or positive serology associated with COVID19 symptoms to be positive for SARS-CoV-2 infections.

Disease activity was assessed according to Safety of Estrogens in Lupus Erythematosus: National Assessment-SLE Disease Activity Index (SELENA-SLEDAI) [9] as well as Physician Global Assessment (PGA) on a 0–3 scale. Disease flares were defined according to the SELENA-SLEDAI flare index as mild/moderate or severe flares. We have considered as patients with severe lupus those with renal and/or neuropsychiatric involvement.

Our sample can be considered representative of a larger population since we included consecutive patients scheduled for visits in our centre.

This study was reviewed and approved by local ethical review board “Comitato Etico Area Vasta Nord Ovest”. Informed consent was obtained from patients orally during telemedicine visits, and it was registered on medical records.

### Statistical analysis

Continuous variables were described as median and 25–75 interquartile range (IQR) or as mean and standard deviation (SD), as appropriate. Categorical variables are reported as proportions. Cross-tabulated data were analysed using Chi-squared test and student’s T-test. Multivariate analyses were performed using the logistic regression model. P values <0.05 were considered to be statistically significant. All the statistical analyses were performed using STATA V.13 software.

## Results

Three hundred and thirty-two patients (303 women) with SLE regularly monitored at our centre were enrolled in this study.

The median age at enrollment was 47 years (IQR 37.3–56), and the median duration of the disease was 17 years (IQR 9–25). Cumulative organ involvement, concomitant medications, symptoms suggesting SARS-CoV-2 infections, cases of quarantine and contacts with confirmed COVID 19 cases of enrolled patients are reported in Table 1.

**Table 1:** Characteristics of the cohort (N = 332).

Contacts with confirmed cases	10 (3.0%)
Mandatory quarantine	14 (4.2%)
Voluntary isolation during lock down period	99 (29.8%)
**Cumulative organ involvement**	**N (%)**
Articular	97 (71.7%)
Muco-cutaneous	198 (59.6%)
Hematological	187 (56.3%)
Renal	144 (43.4%)
Serositis	66 (19.9%)
Neuropsychiatric	31 (9.3%)
Severe lupus	164 (49.4%)
**Symptoms**	
Respiratory symptoms (such as dyspnea or cough)	23 (6.9%)
Fever	25 (7.5%)
Diarrhea	15 (4.5%)
Ageusia/hyposmia or sore throat	10 (3.0%)
Other (such as conjunctivitis)	11 (3.3%)
**Concomitant medications**	
Glucocorticoids	176 (53.0%)
median dose 6-metil prednisolone equivalent	4 mg/day
Hydroxychloroquine	267 (80.4%)
median dose	200 mg/day
Conventional synthetic DMARDs	118 (35.5%)
Mycophenolate	52
Azathioprine	31
Methotrexate	14
Tacrolimus	9
Cyclosporine	7
Leflunomide	4
Sulfasalazine	1
Biological DMARDs	41 (12.4%)
Belimumab	32
Rituximab	6
Abatacept	1
Anakinra	1
Tocilizumab	1
Target synthetic DMARDs	0 (0%)
Other	118 (35.5%)
Combinate therapy with glucocorticoids and at least one DMARDs	96 (28.9%)
Combinate therapy with more than one DMARD	16 (4.8%)

DMARDs: Disease-Modifying Anti-Rheumatic Drugs.

Forty-five (13.5%) patients were screened for SARS-CoV-2 antibodies, and two of them (4.4%; 0.6% of the whole cohort) were confirmed to be positive. Testing for SARS-CoV-2 infection using nasopharyngeal swab was performed in 36 patients (10.9%) and 5 of them (13.9%; 1.5% of the whole cohort) were positive.

Six patients (1.8%) were positive for SARS-CoV-2 infection and their characteristics and course of infections with related therapy are detailed in Table 2.

**Table 2:** Description of cases of COVID-19.

Patient	Age (years)	Cumulative organ involvement	Disease duration (years)	Ongoing treatment at detection of infection	Symptoms	Hospitalization/Treatments for COVID-19	Disease flare during infection period
1	40	Hematological, serositis, articular	4	Low dose of GCs (6MP 4 mg/day), Azathioprine (100 mg/day), HCQ (300 mg/day)	Fever, dyspnea, anosmia, diarrhea	Hospitalization but no ICU admission/Oxygen	No
2	52	Cutaneous, articular, renal	21	Low dose GCs (6MP 4 mg/day), MMF (3000 mg/day), HCQ (400 mg/day)	Cough	Hospitalization but no ICU admission /Increase of GCs dosage (up to 80 mg/day)	No
3	23	Articular, hematological	10	Low dose GCs (6MP 4 mg/day), HCQ (400 mg/day), Belimumab (200 mg/week)	Diarrhea	No hospitalization/No specific therapy	No
4	57	Articular, hematological	30	Low dose GCs (6MP 4mg/day), HCQ (300 mg/day), Belimumab 200 mg/week	Fever, cough, dyspnea, diarrhea	No hospitalization/No specific therapy	No
5	28	Articular, cutaneous, renal, hematological	2	Low dose GCs (6MP 4mg/day), HCQ (200 mg/day)	Asymptomatic	Hospitalization but no ICU admission /No specific therapy	No
6	42	Articular	17	HCQ (200 mg/day), Belimumab 200 mg/week	Fever, cough, dyspnea, diarrhea	No hospitalization/No specific therapy	Yes (articular flare)

GCs: glucocorticoids; HCQ: hydroxychloroquine; ICU: Intensive Care Unit.

Three of these patients (50.0%) required hospitalisation, but none were admitted to an Intensive Care Unit (ICU). Patients 5 was asymptomatic, but her hospitalization was not COVID-19-related.

Two patients were treated with low-flow oxygen, and one of them also with antibiotics and medium-high dose of GCs. All patients continued therapy with HCQ and low dose of GCs, both of which began before the pandemic period. One of the positive patients presented articular flare. No other cases of disease flare were recorded in the other five patients, despite the temporary discontinuation of immunosuppressant or biological drugs, and no deaths were recorded.

We compared characteristics between patients with and without COVID-19 and no differences were observed with regard to age at enrolment, disease duration or type of cumulative organ involvement.

On the other hand, bDMARDs treatment was more frequently assumed by patients with COVID-19 with respect to patients without COVID-19 (50% vs 11.7%; p = 0.005), while no differences were observed for other therapies nor in cases of combined therapies with more DMARDs or GCs and DMARDs. The association between bDMARDs and SARS-CoV-2 didn’t lose significance at multivariate analysis after correction for disease severity (Odds Ratio 7.35, 95% Confidence Interval: 1.42 to 37.87, p = 0.02).

In the whole cohort, 36 patients (11.0%) discontinued at least one drug for SLE as a result of their physician’s advice or personal choice.

During the study period, 27 (8.1%) cases of disease flare were recorded, all of which were mild/moderate. We found a significant correlation between flare and discontinuation of therapies (p<0.001).

In the same period (February-July) of 2019, we assessed 147 patients of the 332 enrolled for this study at the clinic. By comparing the same patients in the two time-frames, we can confirm there is no significant difference in term of incidence of flare (11.6% in 2019 vs 8.8% in 2020).

Data on social isolation were available only for 193 patients. Social isolation required patients to stay at home at all times, avoiding all face-to-face contact outside home. Within this group, 100 were isolated, and flare occurrence was not significantly different versus not isolated patients (7.1% vs 7.9%). COVID-19 infection occurred in 1 isolated and 5 not isolated patients, and the differences did not reach the threshold of statistical significance (p = 0.08).

The results of statistical analysis of characteristics between patients whit COVID-19 and patients without COVID-19, including all p-values for both significant and non-significant results, are reported in Table 3. The results of other statistical analysis are available in S1 Table.

**Table 3:** Statistical analysis of characteristics between patient’s with COVID-19 and patients without COVID-19.

	Patients with COVID-19 (N = 6)	Patients without COVID-19 (N = 326)	P value
Age (years, mean ±SD)	40.3±5.4	47.1±0.7	0.22
Disease duration (years, mean ±SD)	17.7±4.3	17.5±0.6	0.96
Articular involvement (%)	100%	71.2%	0.12
Muco-cutaneous involvement (%)	66.7%	59.5%	0.72
Renal involvement (%)	33.3%	43.6%	0.61
Serositis involvement (%)	16.7%	19.9%	0.84
Neuropsychiatric involvement (%)	16.7%	9.2%	0.51
Glucocorticoids (yes/no) (%)	83.3%	52.45%	0.13
Glucocorticoids daily dose (mean ±SD)	3.6±0.4	4.2±0.2	0.65
Hydroxychloroquine (yes/no) (%)	100%	80.1%	0.22
Conventional synthetic DMARDs (yes/no) (%)	16.7%	35.9%	0.33
Biological DMARDs (yes/no) (%)	50.0%	11.7%	**0.005**
Combinate therapy with more than one DMARD (yes/no) (%)	0%	4.9%	0.57
Combinate therapy with glucocorticoids and at least one DMARDs (yes/no) (%)	50.0%	28.5%	0.25

DMARDs: Disease-Modifying Anti-Rheumatic Drugs.

## Discussion

In our cohort, the incidence of SARS-CoV-2 infection in SLE patients over a six-month period was 1.8%, and the disease course was mild, with no admittance to ICU.

Although a direct comparison is difficult, this was slightly higher than reported in the general Italian or Tuscan population in the same period [10, 11] (1.8% vs 0.4% or 0.3%), but lower compared to COVID-19 cases reported in a SLE northern-Italian cohort (7.2% considering all COVID-19 patients and 2.5% considering only swab-confirmed COVID-19) [1]. In another cohort, just one patient from a group of 397 SLE patients interviewed was positive, but the time-period considered was shorter (patients were contacted between April 9th and April 25th 2020) and none of the patients were screened for SARS-CoV-2 antibodies [12].

Our study suggests that treatment with bDMARDs might have a role in facilitating SARS-CoV-2 infection, however, because this observation is based on a very small group of patients it should be confirmed in larger studies. No other correlation with other treatments was found, including HCQ. It is probable that the concomitant use of multiple immunomodulant and immunosuppressive therapies may have influenced the course of infection in our series, but in a series of 17 patients with SLE under long-term treatment with HCQ this drug does not seems to prevent COVID 19 [2].

With the same aforementioned limitations, our data also suggest that long term GCs use might play a role in SARS-CoV-2 incidence of infection, since 5 of the positive patients were on low dose GCs and it is known that chronic GCs therapy increases risk of viral infections [13]. Similarly, traditional immunosuppressive drugs might have a role, however because of the low numbers we cannot draw any firm conclusions.

Despite the differences did not reach the threshold of statistical significance, it is interesting that the majority of COVID-19 cases occurred in not isolated patients.

The most important finding of this study is that treatment discontinuation seems to be an important cause of disease flare; thus, preventive and abrupt drug withdrawal because of the infectious risk should be avoided or evaluated with caution on a case by case basis considering individual infection risk and comorbidities.

A limitation of our study is that not all patients were screened for SARS-CoV-2 infection and this could bias the incidence of the disease for instance by underestimating asymptomatic patients. Our patients were recruited before a standardized diagnostic screening was established in our country. Therefore, according to working requirements or general practitioner’s prescription, patients were subjected either to swab or serology in non-systematic way.

However, this study represents a real-life setting during the first wave of the health emergency and it addresses the crucial need to balance the need to treat our patients against the risk of infection.

These data are in line with the National and International recommendations on how to manage immunocompromised patients during the pandemic emergency [14, 15] and could be useful to help the treating physician in supporting decision making. In spite of this, patient behaviour concerning adherence to treatment and healthcare attendance is variable. Moreover, this study could serve as encouragement for patients to following their doctor’s advice on treatment and prescriptions in such a difficult situation where fear of risk of infection could lead to counterproductive decisions.

In conclusion, our findings suggest that immunosuppressive treatment should not be preventatively discontinued in SLE patients, but they should be adequately monitored because the infection can mimic disease activity.

## Supporting information

S1 Table Statistical analysis. (DOCX)
