# Supplementary material for: Impact of first wave of SARS-CoV-2 infection in patients with Systemic Lupus Erythematosus: Weighting the risk of infection and flare
Source: PLoS One. 2021 Jan 13;16(1):e0245274. doi: 10.1371/journal.pone.0245274 (PMC7806138; doi:10.1371/journal.pone.0245274)
Supplement: S1 Table — (DOCX) [file pone.0245274.s001.docx]

| **Univariable analysis** | | | | | |
| --- | --- | --- | --- | --- | --- |
| **Variable 1 (% of patients)** | **Variable 2 (% of patients)** | | | **Chi-squared**  **p value** | |
| Cases of disease flare during study period in the whole cohort (8.1%) | Discontinuation of at least one drug in the whole cohort (11.0%) | | | **p<0.001** | |
| Flare in isolated patients in a subgroup of 193 patients (7.1%) | Flare in not isolated patients in a subgroup of 193 patients (7.9%) | | | p=0.819 | |
| COVID-19 infection in isolated patients (0.5%) | COVID-19 in not isolated patients (2.6%) | | | p=0.08 | |
| **Multivariable analysis of predictor of infection of SARS-CoV-2** | | | | | |
| **Infection of SARS-CoV-2** | | **OR** | **P value** | | **95% CI** |
| Biological DMARDs | | 7.34 | **0.02** | | 1.42 to 37.87 |
| Severe lupus | | 0.54 | 0.49 | | 0.09 to 3.09 |

**S1 Table**. **Statistical analysis**
